# Fundus Changes Evaluated by OCTA in Patients With Cerebral Small Vessel Disease and Their Correlations: A Cross-Sectional Study

**DOI:** 10.3389/fneur.2022.843198

**Published:** 2022-04-25

**Authors:** Wang Fu, Xiaoyu Zhou, Minli Wang, Ping Li, Jingjing Hou, Peng Gao, Jue Wang

**Affiliations:** ^1^Department of Neurology, Shanghai Tenth People's Hospital, Tongji University School of Medicine, Shanghai, China; ^2^Department of Ophthalmology, Shanghai Tenth People's Hospital, Tongji University School of Medicine, Shanghai, China; ^3^Tongji University School of Medicine, Shanghai, China; ^4^Educational Office, Shanghai Tenth People's Hospital, Tongji University School of Medicine, Shanghai, China

**Keywords:** retinal capillary density, white matter lesions, perivascular spaces, cerebral microbleeds, optical coherence tomography angiography

## Abstract

**Objective:**

To detect fundus changes in patients with cerebral small vessel disease (CSVD) using optical coherence tomography angiography (OCTA) and to investigate the correlations between CSVD and fundus changes.

**Methods:**

From January 2019 to January 2020, patients diagnosed with CSVD by magnetic resonance imaging (MRI) were enrolled in our study and received fundus examinations using OCTA. CSVD was defined as white matter hyperintensities, enlarged perivascular spaces, lacunes, or microbleeds on MRI. OCTA parameters included foveal avascular zone areas, retinal nerve fiber layer thickness, and capillary densities of the superficial retinal capillary plexuses, deep retinal capillary plexuses, and the radial peripapillary capillary network of the disc. Univariate and multivariate logistic regression analyses were performed to explore the correlation between CSVD and fundus changes.

**Results:**

A total of 115 patients (40% male) were enrolled and analyzed, and the mean age was 65.11 ± 11.23 years. After multivariate logistic regression analysis, the radial peripapillary capillary network density was negatively correlated with severity of deep white matter lesions (OR: 0.909; 95% CI: 0.828–0.998; *p* = 0.046) and perivascular spaces (OR: 0.881; 95% CI: 0.779–0.995; *p* = 0.041). Parafoveal vessel densities of the superficial retinal capillary plexuses were independently correlated with lacunes (OR: 0.889; 95% CI: 0.817–0.967; *p* = 0.006).

**Conclusion:**

OCTA parameters were correlated with CSVD, indicating that OCTA is a potential method for CSVD screening.

## Introduction

Cerebral small vessel disease (CSVD) is commonly used to describe a disorder consisting of clinical, cognitive, neuroimaging, and neuropathological manifestations induced by the brain's small perforating arterioles, capillaries, and perhaps venules (1, 2). CSVD presents with lacunes, cerebral hemorrhage, white matter lesions (WMLs), microbleeds, and microstroke (3). The prevalence of CSVD in older adults (aged > 65 years) is estimated at 87%. Lacunar stroke, which is caused by small vessel disease, accounts for 25–50% of all ischemic strokes in China. Severe WMLs could lead to cognitive decline (vascular dementia), depression, abnormal gait, dyskinesia, dysphagia, and urinary incontinence (4). Hence, early screening for CSVD is of great importance.

The features of CSVD on magnetic resonance imaging (MRI) include *de novo* small subcortical infarcts, lacunes, white matter hyperintensities, enlarged perivascular spaces (PVS), microbleeds, and brain atrophy (5, 6). Currently, MRI is the most commonly used imaging modality in clinical practice to evaluate CSVD. However, the high cost and incompatibility of MRI with certain metallic components prevent its use as a screening method. Therefore, a simpler, more cost-effective screening modality is required. The retina and the optic nerve head are components of the central nervous system (7), and the anatomic, embryologic, and physiologic characteristics of the retinal blood vessels are similar to those of the cerebral small vessels (8–10). The retinal vasculature that is correlated with the development of the cerebral vasculature can be directly visualized with ophthalmoscopy or retinal photography findings and therefore could provide information regarding CSVD (9, 10).

Optical coherence tomography angiography (OCTA) is a novel technique used to optically dissect and visualize the retinal microvascular system through a layer-by-layer analysis. OCTA provides high-resolution images and does not require injection of contrast medium (8, 11, 12) and is thereby a promising potential method to detect CSVD. Previous studies involving OCTA and CSVD have either focused on patients with Alzheimer's disease (AD) or patients with mild cognitive impairment, or were limited to small cohorts (8). Moreover, previous studies have only explored the correlation between WMLs and fundus changes. However, different mechanisms of WMLs have not been discussed in terms of location. Therefore, this comprehensive study included an appropriate sample size to explore the correlation between different CSVD type and fundus changes indicated by OCTA parameters, suggesting the feasibility and effectiveness of OCTA in CSVD screening.

## Materials and Methods

### Participants

From January 2019 to January 2020, patients diagnosed with CSVD on MRI were enrolled. These participants underwent ophthalmic examinations, including slit-lamp, intraocular pressure, fundus photography, and OCTA at Shanghai Tenth People's Hospital, Tongji University. This study was approved by the Ethics Committee of Shanghai Tenth People's Hospital (approval number: 21k253) and complied with the Declaration of Helsinki guidelines. Informed consent was obtained from all participants. Inclusion criteria were as follows: (1) aged ≥ 18 years, (2) underwent brain MRI and OCTA examinations as well as other ophthalmic examinations, (3) have at least one imaging feature of CSVD confirmed by MRI. Exclusion criteria were as follows: (1) history of ocular surgery or primary eye diseases involving the retina or refractive media opacity that hindered observations of the fundus, such as retinal choroid inflammatory diseases, cataracts, glaucoma, posterior vitreous detachment, epiretinal membrane, macular degeneration, optic neuropathies, or severe diabetic or hypertensive retinopathy, (2) contraindications to MRI, (3) unable to undergo OCTA as well as other ophthalmic examinations, (4) AD, (5) diseases such as large-area cerebral infarction, intracerebral hemorrhage, or brain tumor affecting the evaluation of CSVD.

One eye from each enrolled participant was randomly selected. All eligible patients and their medical records were reviewed for demographic, diagnosis, MRI, and follow-up information. Demographic data included age, sex, history of hypertension, diabetes mellitus, and hyperlipidemia. The MRI examination included T1, T2 weighted imaging (T1WI, T2WI), fluid-attenuated inversion recovery (FLAIR) sequence, and T2^*^-weighted gradient-echo. The MRI's were reviewed by a neuroradiologist who was masked to the OCTA findings.

### White Matter Lesions

WMLs were lesions that exhibited hyperintensity on FLAIR and T2WI without obvious hypointensity on T1WI. Periventricular WMLs (PWMLs) were defined if the lesions were adjacent to the ventricle, otherwise, defined as deep WMLs (DWMLs) (13). An experienced neuro-radiologist assessed the Fazekas scores of WMLs using FLAIR. The specific Fazekas scoring was as follows: periventricular hyperintensity was graded as 0 = absence, 1= “caps” or pencil-thin lining, 2 = smooth “halo,” 3 = irregular periventricular hyperintensity extending into the deep white matter. Deep white matter hyperintense signals were defined as 0 = absent, 1 = punctate foci, 2 = beginning confluence of foci, 3 = large confluent areas (13). WMLs were divided into a mild group (0–1 points) and a moderate to severe group (2–3 points) in this study.

### Lacunes

Lacunes were identified as subcortical cavities with fluid between 3 and 15 mm in diameter, located in the territories of perforating arterioles, consistent with a previous acute small deep brain infarct or hemorrhage in the territory of one perforating arteriole (14). They appeared hypointense on T1 and hyperintense on T2, and as a hypointense cavity in the FLAIR sequence, surrounded by a hyperintense halo for reactive gliosis, which was different from PVS (14). In this study, lacunes were dichotomized as presence and absence.

### Perivascular Spaces

PVS were considered as small and well-defined images (< 3 mm in diameter) with a signal intensity equal to that of the cerebrospinal fluid located in the basal ganglia, semioval center, or radiated corona, that followed the course of penetrating vessels; they appeared linear when imaged parallel to the course of the vessel and round or ovoid when imaged perpendicular to the course of the vessel (14). An increase in signal intensity on MRI was equal to cerebrospinal fluid on T2WI images, and hypointensity on T1WI and FLAIR.

The most commonly used score was based on the PVS number, defined by Potter and Doubal: 0 (none), 1(1-10), 2(11-20), 3(21-40), and 4 (>40) (15). In this study, PVS were classified as mild (1–2 points) and moderate to severe (3–4 points).

### Microbleeds

Cerebral microbleeds (CMB) primarily correspond pathologically to hemosiderin-laden macrophages close to arterioles affected by small vessel diseases (16). These microbleeds could be clearly identified on T2^*^-weighted gradient-recalled echo or susceptibility-weighted imaging sequences, appearing as small hypointense lesions which were generally invisible on CT, FLAIR, T1WI, or T2WI MR sequences. We assessed cerebral microbleeds using T2^*^-weighted gradient-recalled echo in our study. In this study, CMB were dichotomized as presence and absence.

### Optical Coherence Tomography Angiography

The RTVue XR Avanti spectral-domain OCT system (Optovue, Inc., Fremont, CA, USA) was used to evaluate the retinal microvasculature, and measurements were obtained using the manufacturer's AngioVue software. Areas in the fovea (6 mm × 6 mm) and around the optic disc (4.5 mm × 4.5 mm) were scanned. Images with poor quality or inadequate signal [signal strength index (SSI) < 50] or an OCTA motion artifact score of three or four were excluded.

The OCTA software was used to record and analyze the enface images of the foveal avascular zone (FAZ), superficial retinal capillary plexuses (SRCP, located between the internal limiting membrane and 10 μm above the inner plexiform layer), and deep retinal capillary plexuses (DRCP, located between 10 μm above the inner plexiform layer and 10 μm below the outer plexiform layers) networks. The macular microvasculature and retinal nerve fiber layer (RNFL) thickness in both the SRCP and DRCP were evaluated. Three concentric circles of macular microvasculature density were analyzed as described in a previous study (17), which included a central circular subfield (disc radius: 0.3 mm), an internal annular zone (0.3–1.5 mm from the fovea; defined as “parafoveal area”) and an external annular zone (1.5–3.0 mm from the fovea, defined as the “perifoveal area”).

The radial peripapillary capillary (RPC) networks, which extended from the internal limiting membrane to the nerve fiber layer, were automatically separated by the instrument software. The RPC density, inside disc microvasculature density, and RNFL thickness were automatically assessed by the OCTA and OCT software.

### Statistical Analysis

Data were collected and analyzed by SPSS version 20.0 for Windows (IBM Co., Armonk, NY, USA). Continuous variables were presented as means ± standard deviations, and categorical variables were presented as frequencies and percentages. Univariate and multivariate logistic analyses were performed to explore the relationship between OCTA parameters and CSVD. On univariate analysis, Student's *t*-test and X^2^ test were used for continuous and categorical variables, respectively. Non-normal distributions were analyzed using the Mann–Whitney *U-*test and presented as medians (interquartile ranges, IQRs). Variables exhibiting significant differences (*p* < 0.05) in the univariate analysis were included in the multivariate logistic model. All *p*-values were two-tailed, and a *p* < 0.05 was defined as statistically significant.

## Results

A total of 132 patients diagnosed with CSVD were screened at our center. Seventeen patients were excluded due to poor OCTA imaging quality. Finally, 115 patients (male: 40%, right eye: 50%) were enrolled and analyzed. The mean age was 65.11 ± 11.23 years. The leading comorbidity was hypertension (59.1%), whereas type 2 diabetes mellitus was the least prevalent (28.7%) (Table 1). The mean RPC network density was 47.34 ± 5.09%, inside disc capillary density was 48.47 ± 4.88%, parafoveal capillary density was 44.37 ± 6.92%, perifoveal capillary density was 45.29 ± 4.61%, and FAZ was 0.34 ± 0.28 mm^2^.

**Table 1:** Univariate analysis of clinical features for patients with CSVD.

	**DWMLs**			**PWMLs**			**PVS**			**Lacunes**			**CMB**		
	**Fazekas 0–1** **(*n* = 62)**	**Fazekas 2–3** **(*n* = 53)**	***p***	**Fazekas 0–1** **(*n* = 62)**	**Fazekas 2–3** **(*n* = 53)**	***p***	**1–2 score** **(*n* = 71)**	**3–4 score** **(*n* = 44)**	***p***	**Yes** **(*n* = 65)**	**No (*n* = 50)**	***p***	**Yes** **(*n* = 75)**	**No** **(*n* = 40)**	***p***
	**No. (%)**			**No. (%)**			**No. (%)**			**No. (%)**			**No. (%)**		
Male	25 (40.3)	21 (39.6)	0.939	27 (43.5)	19 (35.8)	0.401	25 (35.2)	21 (47.7)	0.183	19 (38.0)	27 (41.5)	0.701	14 (35.0)	32 (42.7)	0.424
Hypertension	30 (48.4)	38 (71.7)	0.014	28 (45.2)	40 (75.5)	<0.001	34 (47.9)	32 (72.7)	0.012	22 (44.0)	46 (70.8)	0.004	18 (45.0)	50 (66.7)	0.024
DM	20 (32.3)	13 (24.5)	0.361	22 (35.5)	11 (20.8)	0.082	24 (33.8)	9 (20.5)	0.124	13 (26.0)	20 (30.8)	0.575	7 (17.5)	26 (34.7)	0.053
Hyperlipidemia	29 (53.2)	18 (66.0)	0.164	31 (50.0)	16 (30.2)	0.031	33 (46.5)	14 (31.8)	0.120	17 (34.0)	30 (46.2)	0.189	11 (27.5)	36 (48.0)	0.033
	$\bar{x}$ **±SD**			$\bar{x}$ **±SD**			$\bar{x}$ **±SD**			$\bar{x}$ **±SD**			$\bar{x}$ **±SD**		
Age (years)	61.4 ± 11.2	69.5 ± 9.6	<0.001	61.6 ± 11.3	69.2 ± 9.8	<0.001	62.1 ± 10.0	70.0 ± 11.6	<0.001	66.3 ± 10.6	64.2 ± 11.7	0.323	68.1 ± 8.2	63.5 ± 12.3	0.037

*CSVD, cerebral small vessel disease; DWMLs, deep white matter lesions; PWMLs, paraventricular white matter lesions; PVS, perivascular spaces; CMB, cerebral microbleed; DM, diabetes mellitus; SD, standard deviation*.

**Figure 1:**
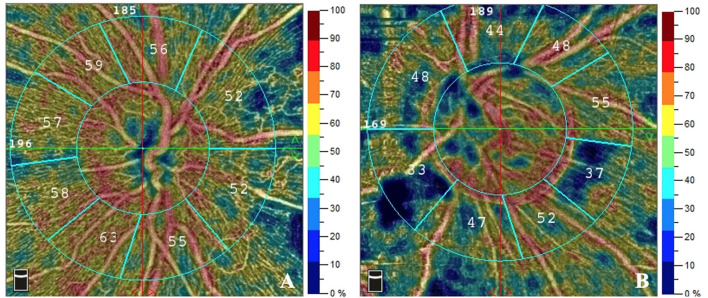
The RPC density of patients with different Fazekas scores of DWMLs. The RPC density evaluated by OCTA was 56.1% in a patient with Fazekas score 1 **(A)** and 45.0% in a patient with Fazekas score 3 **(B)**. RPC, radial peripapillary capillary; DWMLs, deep white matter lesions; OCTA, optical coherence tomography angiography.

Univariate analysis revealed significant correlation between the severity of DWMLs and hypertension (*p* = 0.014), older age (*p* < 0.001), and lower RPC network density (*p* = 0.017) (Table 2). Multivariate logistic regression analysis showed that RPC network density (OR: 0.909; 95% CI: 0.828–0.998; *p* = 0.046) was negatively correlated with the severity of DWMLs (Figure 1). The severity of PWMLs was correlated with hypertension (*p* < 0.001), hyperlipidemia (*p* = 0.031), older age (*p* < 0.001), and lower inside disc capillary density (*p* = 0.080) in univariate analysis. However, no independently correlated variable was found (Table 3).

**Table 2:** Univariate analysis of OCT and OCTA parameters for patients with CSVD.

	**DWMLs**			**PWMLs**			**PVS**			**Lacunes**			**CMB**		
	**Fazekas 0–1 (*****n*** **=** **62)**	**Fazekas 2–3 (*****n*** **=** **53)**	***p***	**Fazekas 0–1 (*****n*** **=** **62)**	**Fazekas 2–3 (*****n*** **=** **53)**	***p***	**1–2 score (*****n*** **=** **71)**	**3–4 score (*****n*** **=** **44)**	***p***	**Yes (*****n*** **=** **65)**	**No (*****n*** **=** **50)**	***p***	**Yes (*****n*** **=** **75)**	**No (*****n*** **=** **40)**	***p***
	$\bar{x}$ **±SD**			$\bar{x}$ **±SD**			$\bar{x}$ **±SD**			$\bar{x}$ **±SD**			$\bar{x}$ **±SD**		
**SRCP**			
Parafoveal capillary density (%)	44.8 ± 6.4	43.8 ± 7.5	0.438	44.5 ± 6.5	44.2 ± 7.5	0.804	44.7 ± 6.4	43.9 ± 7.7	0.545	41.4 ± 7.0	46.7 ± 6.0	<0.001	44.7 ± 8.0	44.2 ± 6.3	0.678
Perifoveal capillary density (%)	45.4 ± 4.4	45.2 ± 4.9	0.827	45.6 ± 4.5	44.9 ± 4.8	0.431	45.4 ± 4.5	45.1 ± 4.9	0.720	44.1 ± 4.6	46.2 ± 4.4	0.015	45.6 ± 4.7	45.1 ± 4.6	0.637
**DRCP**			
Parafoveal capillary density (%)	48.6 ± 7.3	48.2 ± 6.6	0.709	48.5 ± 6.8	48.3 ± 7.3	0.918	48.9 ± 7.2	47.7 ± 6.6	0.361	48.3 ± 7.4	48.5 ± 6.7	0.868	48.0 ±7.3	48.6 ± 6.9	0.643
Perifoveal capillary density (%)	42.3 ± 7.3	42.2 ± 5.0	0.913	42.6 ± 7.0	41.9 ± 5.5	0.609	42.6 ± 7.2	41.8 ± 5.0	0.496	42.0 ± 5.6	42.5 ± 6.8	0.720	41.5 ± 6.6	42.7 ± 6.2	0.388
FAZ (mm^2^)	0.3 ± 0.1	0.4 ± 0.4	0.205	0.3 ± 0.2	0.4 ± 0.4	0.222	0.3 ± 0.1	0.4 ± 0.4	0.577	0.4 ± 0.4	0.3 ± 0.2	0.295	0.4 ± 0.4	0.3 ± 0.2	0.110
Parafoveal RNFL	97.2 ± 18.8	97.2 ± 14.2	0.991	98.3 ± 18.7	96.0 ± 14.3	0.462	97.3 ± 17.0	97.0 ± 16.7	0.934	94.9 ± 16.9	99.0 ± 16.6	0.192	92.8 ± 19.1	99.6 ± 15.0	0.055
thickness (μm)			
Perifoveal RNFL	95.7 ± 11.3	94.2 ± 9.3	0.427	95.5 ± 11.2	94.4 ± 9.5	0.586	95.7 ± 10.4	93.9 ± 10.5	0.393	93.4 ± 12.6	96.3 ± 8.2	0.158	94.5 ± 11.7	95.3 ± 9.7	0.694
thickness (μm)		
RPC density (%)	48.4 ± 4.8	46.1 ± 5.2	0.017	47.6 ± 4.6	47.1 ± 5.6	0.623	48.5 ± 4.2	45.4 ± 5.9	0.001	45.8 ± 5.9	48.5 ± 4.1	0.004	47.2 ± 5.7	47.4 ± 4.8	0.812
Inside disc capillary density (%)	49.3 ± 4.4	47.6 ± 5.2	0.061	49.2 ± 5.2	47.6 ± 4.4	0.080	49.3 ± 4.5	47.1 ± 5.2	0.019	48.2 ± 4.9	48.7 ± 4.9	0.659	46.8 ± 5.0	49.4 ± 4.6	0.007
Peripapillary RNFL thickness (μm)	107.3 ± 10.5	105.9 ± 11.2	0.487	107.1 ± 10.6	106.1 ± 11.1	0.641	108.9 ± 7.8	103.0 ± 13.7	0.004	104.3 ± 12.3	108.5 ± 9.1	0.040	107.4 ± 11.3	106.2 ± 10.6	0.579

*OCT, optical coherence tomography; OCTA, optical coherence tomography angiography; CSVD, cerebral small vessel disease; DWMLs, deep white matter lesions; PWMLs, paraventricular white matter lesions; PVS, perivascular spaces; CMB, cerebral microbleed; SD, standard deviation; SRCP, superficial retinal capillary plexuses; DRCP, deep retinal capillary plexuses; FAZ, foveal avascular zone; RNFL, retinal nerve fiber layer; RPC, radial peripapillary capillary*.

**Table 3:** Multivariate analysis of the related factors for each type of CSVD.

**Variables**	**OR**	**95% CI**	***P***
**DWMLs**			
Age	1.082	1.027–1.140	0.003
Hypertension	1.629	0.671–3.957	0.281
RPC density	0.909	0.828–0.998	0.046
Inside disc capillary density	0.966	0.880–1.059	0.459
**PWMLs**			
Hypertensions	3.019	1.196–7.626	0.019
Age	1.078	1.022–1.136	0.005
DM	1.498	0.556–4.031	0.424
Hyperlipidemia	1.753	0.693–4.432	0.236
Inside disc capillary density	0.999	0.914–1.091	0.975
**PVS**			
Hypertension	1.354	0.526–3.485	0.530
Age	1.078	1.020–1.138	0.007
RPC density	0.881	0.779–0.995	0.041
Inside disc capillary density	0.928	0.836–1.032	0.167
Peripapillary RNFL thickness	0.964	0.907–1.023	0.225
**Lacunes**			
Hypertension	3.156	1.348–7.392	0.008
SRCP-parafoveal capillary density	0.889	0.817–0.967	0.006
SRCP-perifoveal capillary density	1.012	0.892–1.147	0.856
RPC density	0.930	0.831–1.041	0.209
Peripapillary RNFL thickness	0.987	0.931–1.048	0.672
**CMB**			
Age	1.037	0.990–1.086	0.128
Hypertension	1.231	0.479–3.167	0.666
DM	1.633	0.572–4.661	0.359
Hyperlipidemia	1.75	0.672–4.559	0.252
Parafoveal RNFL thickness	0.984	0.959–1.009	0.203
Inside disc capillary density	0.924	0.837–1.020	0.117

*CSVD, cerebral small vessel disease; OR, odds ratio; CI, confidence interval; DWMLs, deep white matter lesions; PWMLs, paraventricular white matter lesions; DM, diabetes mellitus; PVS, perivascular spaces; RPC, radial peripapillary capillary; SRCP, superficial retinal capillary plexuses; CMB, cerebral microbleed; RNFL, retinal nerve fiber layer*.

Patients with lacunes were more likely to be associated with hypertension (*p* = 0.004), lower parafoveal capillary density (*p* < 0.001) and lower perifoveal capillary density (*p* = 0.015) of the SRCP, lower RPC network density (*p* = 0.004) and thinner RNFL (*p* = 0.04) compared to those without lacunes. Multivariate logistic analysis revealed that lower parafoveal capillary density of SRCP (OR: 0.889; 95% CI: 0.817–0.967; *p* = 0.006) and hypertension (OR: 3.156; 95% CI: 1.348–7.392; *p* = 0.008) were independently correlated with lacunes.

Patients in the moderate to severe PVS group had significantly higher blood pressure (*p* = 0.012), older age (*p* < 0.001), lower RPC network density (*p* = 0.001), thinner peripapillary RNFL (*p* = 0.004), and lower inside disc capillary density (*p* = 0.019). After multivariate logistic analysis, RPC network density showed a negative correlation with PVS severity (OR: 0.881; 95% CI: 0.779–0.995; *p* = 0.041).

There were significant differences between CMB patients and non-CMB patients regarding hypertension (*p* = 0.024), hyperlipidemia (*p* = 0.033), age (*p* = 0.037), and inside disc capillary density (*p* = 0.007) in the univariate analysis. However, no variables were significantly different after multivariate logistic regression analysis.

## Discussion

Recently, CSVD has garnered increasing attention, and there has been great demand for a simpler screening method for CSVD in clinical practice. Anatomical, embryologic, and physiological characteristics of retinal blood vessels are similar to those of cerebral small vessels (8–10). The retina is regarded as a direct extension of the diencephalon and shares similar angiogenesis patterns. Meanwhile, vessels in the cerebral cortex (50–200 μm) and those of the retina (50–170 μm) have similar diameters and are approximately equal in size. Physiologically, the retina has a highly isolated and protected vascular system similar to that of the brain (18, 19). In 1986, Hinton et al. (20) described how neuro-degenerative changes affected the retina, lending credence to the correlation between the retina and the brain. Moreover, the fundus is the only part of the body that enables direct observation of nerves and blood vessels, thereby providing a direct path reflecting the nerves and blood vessels of the brain.

OCTA, a non-invasive imaging technique that provides high-resolution volumetric data, has been widely used in clinical practice. However, OCTA has not been applied to evaluate the correlation between fundus vessels and CSVD. In 2018, Lahme et al. analyzed 74 patients with AD and demonstrated that the Fazekas score was significantly correlated with retinal flow density rather than the area of the FAZ. However, RNFL thickness was not included in their study (21). Further, den Haan et al. (14) enrolled patients with AD and found that vessel density in the outer ring of the macula was inversely associated with the Fazekas score, whereas no correlation was identified between the RNFL thickness or FAZ and the Fazekas score. Together, these studies focused on patients with AD or mild cognitive impairment, and the sample size was small. To date, the application of OCTA in the evaluation of CSVD is lacking. We conducted this study to explore the correlation between CSVD and fundus changes revealed by OCTA.

Patients with lower RPC density had a higher Fazekas score in DWMLs rather than in PWMLs. There are some possible explanations: (1) as indicated by histological findings, PWMLs are non-ischemic. However, microcystic infarcts and patchy rarefaction of myelin which are ischemic, are associated with DWMLs (22–24); (2) anatomically, the paraventricular white matter area might be more hemodynamically determined, because this area is supplied by non-collateralizing ventriculofugal vessels arising from the subependymal arteries originating from the choroidal arteries, or from the terminal branches of the ramistriate and ventriculofugal vessels that run toward the penetrating centripetal vessels coming from the pial surface supply. Hence, the blood supply is not solely from small cerebral vessels in the periventricular white matter areas (24, 25). Conversely, deep white matter is supplied by medullary arteries arising from the cortical branches of middle cerebral arteries and has a single blood supply system, thus being more susceptible to ischemia (26).

Our study revealed a close inverse correlation between the RPC density and the Fazekas score in DWMLs. However, no correlation was detected between the Fazekas score and capillary density of the inside disc or macula. We propose a possible reason is that there are two distinct circulatory systems within the disc, the central retinal artery (CRA) and the ciliary arteries, whereas the radial peripapillary of the disc is only supplied principally by recurrent retinal arterioles arising from branches of the CRA. As a result, the radial peripapillary of the disc is more susceptible to ischemia.

In Lahme's study, the correlation between the vessel density of the RPC and the Fazekas score of WMLs was also demonstrated, which is in agreement with the findings in our study (21). However, they also reported that the vessel density of the parafoveal was related to the Fazekas score, which differed from our results. This is possibly because their study focused on patients with AD; the sample size could have also contributed to differences in the results obtained.

The present study also revealed that the lower density of RPCs was not only related to DWMLs, but the severity of PVS. Doubal et al. (27) reported that enlargement of the PVS was associated with WMLs. The PVS serves as an important conduit for the drainage of interstitial fluid to the ventricles, and it could be affected by abnormalities of the blood-brain barrier (28). Therefore, the parallel formation mechanism between WMLs and PVS may lead to similar changes in the fundus and comparable trends in RPC density, which could explain our results (29).

Lacunes are caused by small vessel disease, and pathological vascular changes in lacunes were mainly lipid hyaline degeneration or micro atherosclerotic plaque (30). As reported previously, our study also indicated patients with lacunes exhibited lower vessel density of the parafoveal SRCP (14, 21). However, the underlying mechanism requires further exploration. We suspected that the possible reason might be that the vascular density of the parafovea was lower than that of the perifovea in healthy individuals; therefore, the parafovea may be more vulnerable to ischemia.

No independent relationship was established between deep vascular density or the thickness of the fibrous layer and lacunes. Within the retina, there were two distinct circulatory systems, the superficial retina was supplied by the CRA, and the deep retina was supplied by ciliary arteries. Unlike the ciliary arteries, the CRA is a terminal vessel. Animal studies have demonstrated that ciliary arteries transport a greater volume of blood than the retinal the blood flow (31, 32). Therefore, the deep retina with its greater blood supply was less affected by ischemia than the superficial, which may account for the lack of significant correlation between the deep vascular densities and lacunes. Further, no significant correlation was found between the thickness of the fibrous layer and lacunes, possibly because the fibrous layer is not affected in the early stage of the disease.

There were no significant differences in the parameters of the fundus between patients with and without CMB in this study. The mechanism of CMB is primarily amyloid deformation rather than ischemia, which could explain the results.

There are limitations in this study. First, this is a non-randomized study and a control group was not included. Second, although the sample size is relatively large, studies with even larger cohorts are required to confirm our conclusions. Nevertheless, our study provides practical and useful information regarding the application of OCTA in CSVD screening.

## Conclusion

This study investigated the correlation between four types of cerebrovascular diseases and fundus changes revealed by OCTA in a relatively large cohort. To the best of our knowledge, this is the first study to simultaneously assess four types of CSVD and to analyze PWMLs and DWMLs in parallel. The correlation between fundus parameters and CSVD suggests that OCTA is a feasible screening method for CSVD. Further studies using OCTA for CSVD screening are required to confirm our findings. OCTA parameters correlated with CSVD, with the most important being the RPC network density, which was negatively correlated with the severity of DWMLs and PVS, indicating that OCTA is a promising potential method for CSVD screening.

## Data Availability Statement

The raw data supporting the conclusions of this article will be made available by the authors, without undue reservation.

## Ethics Statement

This study was approved by the Ethics Committee of Shanghai Tenth People's Hospital (No. 21k253). The patients/participants provided their written informed consent to participate in this study. Written informed consent was obtained from the individual(s) for the publication of any potentially identifiable images or data included in this article.

## Author Contributions

WF, XZ, PG, and JW: conception and design. WF, XZ, MW, PL, PG, and JW: analysis and interpretation. WF, XZ, MW, PL, and JH: data collection. WF, XZ, and PG: writing the manuscript. WF, XZ, MW, PL, JH, PG, and JW: final approval of the article and overall responsibility. WF, XZ, PL, JH, PG, and JW: statistical analysis. JW: obtained funding. All authors contributed to the article and approved the submitted version.

## Funding

This work was supported by Shanghai Municipal Key Clinical Specialty (shslczdzk06102), Shanghai Science and Technology Commission (19401972804), and Start-up Fund of Shanghai Tenth People's Hospital (04.03.16.002).

## Conflict of Interest

The authors declare that the research was conducted in the absence of any commercial or financial relationships that could be construed as a potential conflict of interest.

## Publisher's Note

All claims expressed in this article are solely those of the authors and do not necessarily represent those of their affiliated organizations, or those of the publisher, the editors and the reviewers. Any product that may be evaluated in this article, or claim that may be made by its manufacturer, is not guaranteed or endorsed by the publisher.
